# Mathematical Modelling of Bacterial Meningitis Transmission Dynamics with Control Measures

**DOI:** 10.1155/2018/2657461

**Published:** 2018-03-27

**Authors:** Joshua Kiddy K. Asamoah, Farai Nyabadza, Baba Seidu, Mehar Chand, Hemen Dutta

**Affiliations:** ^1^Department of Mathematics, Kwame Nkrumah University of Science and Technology, Kumasi, Ghana; ^2^African Institute for Mathematical Sciences, Biriwa, Ghana; ^3^Division of Mathematics, Stellenbosch University, Western Cape, South Africa; ^4^Department of Mathematics, University for Development Studies, Navrongo, Ghana; ^5^Department of Applied Sciences, Guru Kashi University, Bathinda, India; ^6^Department of Mathematics, Gauhati University, Guwahati 781014, India

## Abstract

Vaccination and treatment are the most effective ways of controlling the transmission of most infectious diseases. While vaccination helps susceptible individuals to build either a long-term immunity or short-term immunity, treatment reduces the number of disease-induced deaths and the number of infectious individuals in a community/nation. In this paper, a nonlinear deterministic model with time-dependent controls has been proposed to describe the dynamics of bacterial meningitis in a population. The model is shown to exhibit a unique globally asymptotically stable disease-free equilibrium *ℰ*_0_, when the effective reproduction number *ℛ*_VT_ ≤ 1, and a globally asymptotically stable endemic equilibrium *ℰ*_1_, when *ℛ*_VT_ > 1; and it exhibits a transcritical bifurcation at *ℛ*_VT_ = 1. Carriers have been shown (by Tornado plot) to have a higher chance of spreading the infection than those with clinical symptoms who will sometimes be bound to bed during the acute phase of the infection. In order to find the best strategy for minimizing the number of carriers and ill individuals and the cost of control implementation, an optimal control problem is set up by defining a Lagrangian function *L* to be minimized subject to the proposed model. Numerical simulation of the optimal problem demonstrates that the best strategy to control bacterial meningitis is to combine vaccination with other interventions (such as treatment and public health education). Additionally, this research suggests that stakeholders should press hard for the production of existing/new vaccines and antibiotics and their disbursement to areas that are most affected by bacterial meningitis, especially Sub-Saharan Africa; furthermore, individuals who live in communities where the environment is relatively warm (hot/moisture) are advised to go for vaccination against bacterial meningitis.

## 1. Introduction

Meningitis is an inflammation of the meninges which are membranes that surround the spinal cord and the brain [1]. It is often caused by viruses, bacteria, and protozoa. Bacterial meningitis is common in children and young adults. This disease mostly spreads in communities/societies that live in close quarters (e.g., police staff, police cells, college students, military staff, and prisons) [2]. Bacterial meningitis is generally caused by germs such as* Listeria monocytogenes*,* Streptococcus pneumoniae*, Group B* Streptococcus*,* Neisseria meningitidis*, and* Haemophilus influenzae*, which spreads from one person to another [3]. This infection varies by age groups: Group B* Streptococcus*,* Streptococcus pneumoniae*,* Listeria monocytogenes*, and* Escherichia coli* are mostly found in newborn babies;* Streptococcus pneumoniae*,* Neisseria meningitidis*,* Haemophilus influenzae* type b (Hib), and Group B* Streptococcus* are common in babies and children;* Neisseria meningitidis* and* Streptococcus pneumoniae* are predominant in teens and young adults; and* Streptococcus pneumoniae*,* Neisseria meningitidis*,* Haemophilus influenzae* type b (Hib), Group B* Streptococcus*, and* Listeria monocytogenes* are commonly found in older adults [3]. Bacterial meningitis is characterized by intense headache and fever, vomiting, sensitivity to light, and stiff neck, which result in convulsion, delirium, and death.

It is estimated that meningococcal meningitis causes over 10,000 deaths annually in Sub-Saharan Africa [4]. About 4,100 cases of bacterial meningitis occurred between 2003 and 2007 in the United States [3, 5]. Between 5% to 40% of children and 20% to 50% of adults with this condition die [6]. Infections from bacterial meningitis can cause permanent disabilities such as brain damage, hearing loss, and learning disabilities [3]. The illness of bacterial meningitis becomes worse when symptoms are not detected early enough; even with proper treatment, the individual could die [2].

Prevention of bacterial meningitis can be achieved through vaccination and/or preventing contact with infectious individuals. Vaccination is the most effective way of protecting children against certain types of bacterial meningitis [3]. Vaccines that can prevent meningitis include* Haemophilus influenza* type B (Hib), pneumococcal conjugate, and meningococcal vaccine [6, 7]. The conjugate meningitis A vaccine, MenAfrivac, is recommended to protect people in Sub-Saharan Africa against the most common type, serotype A [8]. In the United States, the primary means of preventing meningococcal meningitis is antimicrobial chemoprophylaxis [9]. Empirical therapy includes ceftriaxone or cefotaxime and vancomycin for* Streptococcus pneumoniae* [2]. There is a vaccine against meningococcal disease which is 85%–100% effective in preventing four kinds of bacteria (serogroups A, C, Y, and W-135) that cause about 70% of the disease in the United States [2].

Trotter and Ramsay [10] outlined some recommendations on the use of conjugate vaccines in Europe based on the experience with meningococcal C conjugate (MCC) vaccines. In areas with limited health infrastructure and resources, there are a number of antibiotics including penicillin, ampicillin, and chloramphenicol that can be used to treat the infection meningitis.

Mathematical models have been shown to help increase the understanding of the spread and control of infectious diseases. Martínez et al. [2] studied the spread of meningococcal meningitis with the use of a discrete mathematical model, based on cellular automata where the population was divided into five classes: susceptible, asymptomatic infected, infected with symptoms, carriers, recovered, and died classes. Broutin et al. [11] studied the dynamics of meningococcal meningitis in nine African countries by adopting some mathematical tools to time series analysis and wavelet method, the results of their studies suggest that “international cooperation in Public Health and cross disciplines studies are highly recommended to help in controlling this infectious disease.” Miller and Shahab [12] studied the cost effectiveness of immunisation strategies for the control of epidemic meningococcal meningitis. The research work in [13] gives a detailed description of the use of antibiotics for the prevention and treatment of meningitis infection. Irving et al. [14] used deterministic compartmental models to investigate how well simple model structures with seasonal forcing were able to qualitatively capture the patterns of meningitis infection. They demonstrated that the complex and irregular timing of epidemics could be caused by the interaction of temporary immunity conferred by carriage of the bacteria together with seasonal changes in the transmissibility of infection. Actually, there have been a significant number of studies of various types of Meningitis in Africa and Europe without the use of optimal control analysis (see [15–28]).

It is obvious that mathematical modelling has become crucial in investigating the epidemiological behaviour of meningitis. Furthermore, mathematical modelling helps to identify the risk factors for diseases, so as to find out why everyone does not have the same infection uniformly [29].

The application of optimal control in disease modelling gives valuable information on how to apply control measures. Through vaccination, treatment, public education, and so forth, many infectious diseases have been controlled [29]. Since the introduction of optimal control theory in disease modelling, there have been a considerable number of studies of infectious diseases using optimal control analysis (see [30–41]). With the significant influence of optimal control theory in disease modelling, this paper presents an optimal control model for bacterial meningitis in the presence of vaccination and treatment due to public health education. The model is qualitatively analyzed and numerically simulated in order to help give policy direction on how to control the spread of the disease.

The rest of the paper is organized as follows.  Section 2 presents the model formulation and analysis.  Section 3 presents the analysis of the optimal control problem, leading to the existence and characterization of the control measures.  Section 4 contains the numerical simulations and discussion.  Section 5 presents the conclusion of the study.

## 2. Model Formulation and Analysis

**Figure 1:**
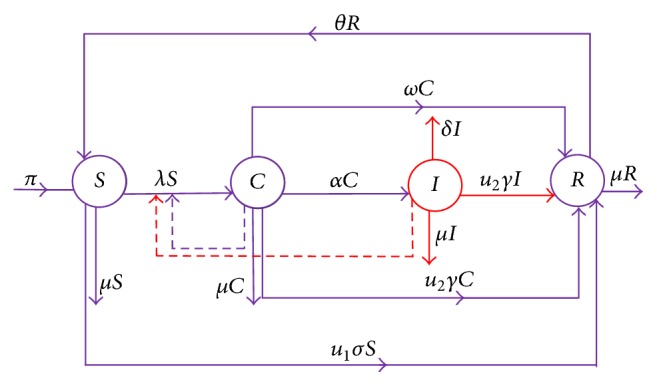
The flowchart diagram describing bacterial meningitis transmission dynamics within the population. The four circles represent the four compartments of individuals, the movement between the compartments is indicated by the continuous arrows, *u*_1_ is a control measure (vaccination), and *u*_2_ is the second control measure (treatment), with the consideration that both control measures lies in 0 < *u*_1_  and  *u*_2_ ≤ 1.

Adopting the epidemiological studies of a meningitis model as presented in [4], we consider four mutually exclusive compartments to indicate individuals with unique natures (i.e., susceptibles, *S*(*t*), carriers, *C*(*t*), ill individuals, *I*(*t*), and recovered individuals, *R*(*t*)) in relation to the disease. It is assumed that the susceptible compartment, *S*(*t*), is populated through recruitment at the rate, *π* (thus migration and/or birth rate), and *β* is the rate of effective contact of carriers and/or infected (ill) individuals in the susceptible population. The carrier compartment consists of individuals that have the infection and do not show any clinical symptoms but contribute to the spread of the disease. When a susceptible individual is exposed to this infection, that individual can harbour the bacterium for weeks or even months [42]; but, in a normal circumstance, an individual develops symptoms of the infection within 3 to 7 days after exposure [3]. Carriers are assumed to develop clinical symptoms (i.e., move to ill individuals compartment, *I*(*t*)) at rate *α*. Ill individuals who are seriously infected are assumed to have no natural recovery except when given treatment on time. From an epidemiological perspective, individuals in the removed/recovered compartment, *R*(*t*), do not attain permanent immunity. After vaccination, immunity develops within 7–10 days and remains effective for approximately 3–5 years [2]. Therefore, it is assumed that the immunity acquired from developing the diseases or carrying the bacteria or through vaccination is of the same intensity (they all lead to the recovered compartment from which people return to the susceptible compartment at a given unique rate *θ*). Research indicates that carriers may recover naturally from the infection without treatment, and we denote such natural recovery rate as *ω*. Infected individuals are assumed to die from disease at rate *δ*. Since natural death is inevitable, *μ* is assumed to be the natural death rate of individuals in all the compartments. Vaccination and treatment due to public health education have been shown to be strategies of control of diseases. Therefore we introduced this two control measures in the model as *u*_1_(*t*) and *u*_2_(*t*), respectively, here *u*_1_; thus vaccination is comprised of both reactive vaccination and preventative vaccination. The effectiveness of both control measures in minimizing the disease is denoted by *σ* and *γ*, respectively. If *σ* = *γ* = 0, it signifies that vaccination and treatment have no effect on the model; if *σ* = *γ* = 1, it also signifies that vaccination and treatment are perfectly effective (i.e., 100% effectiveness) (see [43]). If 0 < *σ*  and  *γ* < 1, it signifies that both vaccine and treatment are imperfect [44]. In view of this, it is assumed that administering treatment to the ill individuals leads to recovery at rate *γu*_2_. It is also assumed that the vaccinated individuals develop partial immunity at rate *σu*_1_. From an epidemiological perspective treatment is not given to carriers in real life (since we do not know who carries the bacteria or not), but it is assumed that carriers of meningitis are just like people with the HIV virus who do not know their status unless they go for medical test; hence this paper seeks to encourage individuals to go for regular test of this bacterial disease; therefore it is assumed that a certain portion of carriers could be treated before any symptoms of the infection show up, which results in *γu*_2_*C* as shown in Figure 1. The vaccine is assumed to be imperfect and thus has a failure rate of (1 − *u*_1_*σ*). Therefore the force of new infections is given by

(1) $$\begin{array}{c} \lambda =\frac{\beta \left(1-u_{1}\sigma \right)\left(\eta C+\eta_{1}I\right)}{N}. \end{array}$$

Equation (1) is often referred to as standard incidence rate of new infections, which is normalized by the total population *N* = *S* + *C* + *I* + *R*.  Table 1 gives a full description of parameters used in the model.

The set of differential equations and flow diagram corresponding to the bacterial meningitis dynamics and disease pathway with control terms is given in system (2) and Figure 1.

(2) dSdt=π−1−u1σβSηC+η1IN−μ+u1σS+θR,dCdt=1−u1σβSηC+η1IN−α+ω+μ+u2γC,dIdt=αC−δ+μ+u2γI,dRdt=u2γI+ω+u2γC+u1σS−θ+μR,S0>0,  C0≥0,  I0≥0,  R0≥0.

Adding the equations in model (2) gives the rate of change of total population as

(3) $$\begin{array}{c} \frac{dN}{dt}=\pi -\mu N-\delta I\leq \pi -\mu N. \end{array}$$

Let

(4) $$\begin{array}{c} \Omega =\left\{\;\left(\;S,C,I,R\;\right)\in R_{+}^{4}:N\leq \frac{\pi }{\mu }\;\right\}. \end{array}$$

System (2) is well-posed with all solutions in *Ω* remaining in *Ω* if initial conditions are positive. It can easily be shown that if the initial conditions start outside *Ω* the solutions tend to *Ω*.

### 2.1. Equilibrium Points

To obtain the equilibrium points of system (2), we assume that the control measures are time-independent (see [45] for similar analysis).

#### 2.1.1. Disease-Free Equilibrium *ℰ*_0_

To obtain the disease-free equilibrium, *C*(*t*),  *I*(*t*), and the right-hand-side of system (2) are set to zero. If susceptible individuals are assumed to receive vaccination against the disease at a constant rate, then the disease-free equilibrium will be given by

(5) E0S0,C0,I0,R0=πθ+μμu1σ+θ+μ,0,0,πu1σμu1σ+θ+μ.

#### 2.1.2. Effective Reproduction Number *ℛ*_VT_

Using the next generation matrix method [46], the effective reproduction number of the bacterial meningitis model with vaccination and treatment is obtained as

(6) $$\begin{array}{c} R_{\text{VT}}=R_{C_{\text{VT}}}+R_{I_{\text{VT}}}, \end{array}$$

where

(7) RCVT=βη1−u1σθ+μα+ω+μ+u2γθ+μ+u1σ,RIVT=βαη11−u1σθ+μα+ω+μ+u2γδ+μ+u2γθ+μ+u1σ.

**Figure 2:**
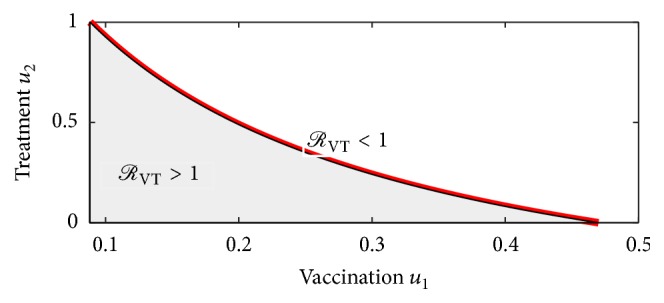
Region where *ℛ*_VT_ < 1 and *ℛ*_VT_ > 1 in the *u*_1_ − *u*_2_ parameter space, with the parameter values *β* = 0.88, *η* = 0.2, *ω* = 0.06, *δ* = 0.03, *θ* = 0.0839, *α* = 0.05, *γ* = 0.2  and  *σ* = 0.7.

To determine how the two control measures impact on the reproduction number, we make a plot of *ℛ*_VT_, on the *u*_1_ − *u*_2_ plane for arbitrary constant values of model parameters in Figure 2. From the figure, it is shown that *u*_2_ decreases with increasing *u*_1_. So an increase in vaccination levels decreases the need for treatment. Figure 2 also shows the region in which the vaccination and treatment values should lie for the disease control.

#### 2.1.3. Endemic Equilibrium *ℰ*_1_

System (2) can be shown to have a unique endemic equilibrium of the form (*S*^*∗*^, *C*^*∗*^, *I*^*∗*^, *R*^*∗*^), where

(8) S∗=απμ+θ+ω+u2γδ+μ+u2γθ+θαu2γ−α+ω+μ+u2γδ+μ+u2γθ+μπαRVT−1αμθ+μ+u1σARVT−1+B,C∗=δ+μ+u2γπαRVT−1αARVT−1+B,R∗=απu1σ+δ+μ+u2γω+u2γμ+u1σ+αu2γμ+u1σ−α+ω+μ+u2γδ+μ+u2γu1σπαRVT−1αμθ+μ+u1σARVT−1+B,I∗=παRVT−1ARVT−1+B,

with **A** = (*δ* + *μ* + *u*_2_*γ*)(*α* + *ω* + *μ* + *u*_2_*γ*) and **B** = (*δ* + *μ* + *u*_2_*γ*)[*ω* + *u*_2_*γ* − *u*_1_*σ*) + *α*(*u*_2_*γ* + *μ*)].

**Remark 1 .**

(i) If *ℛ*_VT_ < 1, then system (2) will have only one equilibrium: the disease-free equilibrium.

(ii) If *ℛ*_VT_ > 1, then system (2) will have two equilibria: the disease-free equilibrium, *ℰ*_0_, and the endemic equilibrium, *ℰ*_1_.

(iii) The case *ℛ*_VT_ = 1 is a critical threshold point where the disease-free equilibrium *ℰ*_0_ loses its local asymptotic stability. Thus *ℛ*_VT_ = 1 gives the idea of transcritical bifurcation where the stability of system (2) moves between *ℰ*_0_ and *ℰ*_1_ [37].

### 2.2. Stability Analysis

In analyzing the local stability of the disease-free equilibrium, the Routh-Hurwitz criteria are used, and, for the global stability of the two equilibria, the direct Lyapunov technique is employed.

#### 2.2.1. Local Stability Analysis of *ℰ*_0_

**Theorem 2 .**

The disease-free equilibrium of system (2) is locally asymptotically stable if *ℛ*_VT_ < 1 and unstable if *ℛ*_VT_ > 1.

**Proof**

Evaluating the Jacobian matrix of system (2) at the disease-free equilibrium gives

(9) $$\begin{array}{c} J\left(\;E_{0}\;\right)=\left[\begin{array}{cccc} -\left(\mu +u_{1}\sigma \right) & -\frac{\left(1-u_{1}\sigma \right)\beta \eta \left(\theta +\mu \right)}{\left(u_{1}\sigma +\theta +\mu \right)} & -\frac{\left(1-u_{1}\sigma \right)\beta \eta_{1}\left(\theta +\mu \right)}{\left(u_{1}\sigma +\theta +\mu \right)} & \theta \\ 0 & \frac{\left(1-u_{1}\sigma \right)\beta \eta \left(\theta +\mu \right)}{\left(u_{1}\sigma +\theta +\mu \right)}-\left(\alpha +\omega +\mu +u_{2}\gamma \right) & \frac{\left(1-u_{1}\sigma \right)\beta \eta_{1}\left(\theta +\mu \right)}{\left(u_{1}\sigma +\theta +\mu \right)} & 0\\ 0 & \alpha & -\left(\delta +\mu +u_{2}\gamma \right) & 0\\ u_{1}\sigma & \left(\omega +u_{2}\gamma \right) & u_{2}\gamma & -\left(\theta +\mu \right) \end{array}\right]. \end{array}$$

The characteristic polynomial of the Jacobian matrix *J*(*ℰ*_0_) is given by

(10) $$\begin{array}{c} P\left(\;\lambda \;\right)=\lambda^{4}+DT\lambda^{3}+DT_{1}\lambda^{2}+DT_{2}\lambda +DT_{3}, \end{array}$$

where

(11) T=A+B+C−RCVT,T1=A+B−RCVT2μ+u1σ+θ+μ+u1σθ+μ+μθ1−RVT,T2=θ+μ1−RVT+A−RCVT−RCVT+μ+u1σ1−RIVT+δ+μ+u2γθ+μ+A+B−RCVTμθ,T3=θμRIVT−1+μ+u1σθ+μRIVT−1,A=1δ+μ+u2γ,B=1α+ω+μ+u2γ,C=μ+u1σ+θ+μα+ω+μ+u2γδ+μ+u2γ,D=α+ω+μ+u2γδ+μ+u2γ.

The Routh-Hurwitz conditions [46] that guarantee that the eigenvalues of the characteristic polynomial in (10) have negative real parts are given by

(12) DT>0,DT1>0,DT2>0,DT3>0,D3TT1T2>D2T22+D3T2T3.

These conditions are easily seen to be satisfied when *ℛ*_VT_ < 1. Thus, the disease-free equilibrium of system (2) is locally asymptotically stable when *ℛ*_VT_ < 1 and unstable when *ℛ*_VT_ > 1. This completes the proof.

#### 2.2.2. Global Stability of *ℰ*_0_

**Theorem 3 .**

The disease-free equilibrium *ℰ*_0_ of system (2) is globally asymptotically stable if *ℛ*_VT_ ≤ 1 and unstable if *ℛ*_VT_ > 1.

**Proof**

Let *𝒱*(*S*, *C*, *I*, *R*), with positive constants, *𝒦*_1_ and *𝒦*_2_, be a Lyapunov function defined as

(13) VS,C,I,R=S−S0−S0ln⁡SS0+K1C+K2I+R−R0−R0ln⁡RR0.

Taking the time derivative of the Lyapunov function we obtain

(14) dVdt=1−S0SdSdt+K1dCdt+K2dIdt+1−R0RdRdt,where  K1≥0,  K2≥0.

Substituting *dS*/*dt*,  *dC*/*dt*,  *dI*/*dt*, and *dR*/*dt* in (2) into (14) gives

(15) dVdt=1−SS0π−1−u1σβSηC+η1IN−μ+σu1S+θR+K11−u1σβSηC+η1IN−α+ω+μ+u2γC+K2αC−δ+μ+u2γI+1−RR0u2γI+ω+u2γC+σu1S−θ+μR≤K11−u1σβηθ+μu1σ+θ+μC+1−u1σβη1θ+μu1σ+θ+μI−α+ω+μ+u2γC+K2αC−δ+μ+u2γI.

Since

(16) S≤S0=πθ+μμu1σ+θ+μ,R≤R0=πu1σμu1σ+θ+μ,N≤πμon  Ω,

this implies that

(17) dVdt≤1−u1σβηθ+μu1σ+θ+μK1+αK1−α+ω+μ+u2γK2C+1−u1σβη1θ+μu1σ+θ+μK1−δ+μ+u2γK2I.

Equating the coefficient of *I* in (17) to zero gives

(18) 1−u1σβθ+μK1=δ+μ+u2γu1σ+θ+μK2.

Choosing *𝒦*_1_ = (*δ* + *μ* + *u*_2_*γ*)(*u*_1_*σ* + *θ* + *μ*)  and  *𝒦*_2_ = (1 − *u*_1_*σ*)*β*(*θ* + *μ*) and plugging *𝒦*_1_ and *𝒦*_2_ into (17), we have

(19) dVdt≤α+ω+μ+u2γδ+μ+u2γu1σ+θ+μ·RVT−1C≤0,if  RVT≤1.

Additionally *d𝒱*/*dt* = 0 if and only if *C* = 0. Hence, the largest compact invariant set in {(*S*, *C*, *I*, *R*) ∈ *Ω* : *d𝒱*/*dt* ≤ 0} is the singleton set {*ℰ*_0_}. Therefore, from LaSalle's invariance principle, we conclude that *ℰ*_0_ is globally asymptotically stable in *Ω* if *ℛ*_VT_ ≤ 1 [37, 49].

#### 2.2.3. Global Stability of *ℰ*_1_

**Theorem 4 .**

The endemic equilibrium *ℰ*_1_ of system (2) is globally asymptotically stable whenever *ℛ*_VT_ > 1.

**Proof**

Suppose *ℛ*_VT_ > 1, and then the existence of the endemic equilibrium point is assured. Using the common quadratic Lyapunov function

(20) $$\begin{array}{c} V\left(\;x_{1},x_{2},\ldots ,x_{n}\;\right)=\overset{n}{\underset{i=1}{\sum }}\frac{c_{i}}{2}{\left(\;x_{i}-x_{i}^{*}\;\right)}^{2}, \end{array}$$

as illustrated in [50], we consider the following candidate Lyapunov function:

(21) VS,C,I,R=12S−S∗+C−C∗+I−I∗+R−R∗2.

The time derivative of *𝒱*(*S*, *C*, *I*, *R*) in (21) is given by

(22) dVdtS,C,I,R=S−S∗+C−C∗+I−I∗+R−R∗dS+C+I+Rdt.

Plugging the equations in system (2) into (22) yields

(23) dVdt=S−S∗+C−C∗+I−I∗+R−R∗·π−μS+C+I+R−δI.

Now setting

(24) $$\begin{array}{c} \pi =\mu \left(\;S^{*}+C^{*}+I^{*}+R^{*}\;\right)+\delta I^{*}, \end{array}$$

we have

(25) dVdt=S−S∗+C−C∗+I−I∗+R−R∗·μS∗+C∗+I∗+R∗+δI∗−μS+C+I+R−δI=S−S∗+C−C∗+I−I∗+R−R∗−μS−S∗−μC−C∗−μI−I∗−μR−R∗−δI−I∗.

Further simplification gives

(26) dVdt=−μS−S∗2−μS−S∗·C−C∗+I−I∗+R−R∗+δI−I∗−μC−C∗2−μC−C∗·S−S∗+I−I∗+R−R∗+δI−I∗−μ+δI−I∗2−μI−I∗·C−C∗+R−R∗−μR−R∗2−μR−R∗·S−S∗+C−C∗+I−I∗+δI−I∗.

It has therefore been shown that *d𝒱*/*dt* is negative, and additionally at *ℰ*_1_ (i.e., if *S* = *S*^*∗*^, *C* = *C*^*∗*^, *I* = *I*^*∗*^, and *R* = *R*^*∗*^), *d𝒱*/*dt* = 0. It follows from LaSalle's invariant principle [51] that all solutions of system (2) approach *ℰ*_1_ as *t* → *∞* if *ℛ*_VT_ > 1. Therefore, the endemic equilibrium *ℰ*_1_ is globally asymptotically stable in *Ω* whenever *ℛ*_VT_ > 1 [37, 49]. This completes the proof.

### 2.3. Sensitivity Analysis

Sensitivity analysis is used to determine the response of a model to variations in its parameter values. In the present case, the focus is given to determining how changes in the model parameters impact the effective reproduction number. This is done through the normalized forward-sensitivity index. We also use the Latin hypercube sampling and the partial rank correlation coefficients (PRCC) to plot scatter diagrams and Tornado plots to determine the relative importance of the parameters in *ℛ*_VT_ for the disease transmission and prevalence (see also [52]).

**Definition 5 .**

The normalized forward-sensitivity index of *ℛ*_VT_ to any parameter, say *ρ*, as given in [46] can be defined as

(27) $$\begin{array}{c} \Gamma_{R_{\text{VT}}}^{\rho }=\frac{\partial R_{\text{VT}}}{\partial \rho }\frac{\rho }{R_{\text{VT}}}. \end{array}$$

The sensitivity indexes of *ℛ*_VT_ with respect to its parameters are computed as follows:

(28) ΓRVTβ=∂RVT∂ββRVT=1,ΓRVTη=∂RVT∂ηηRVT=δ+μ+u2γηηδ+μ+u2γ+η1α,ΓRVTη1=∂RVT∂η1η1RVT=αη1αη1+δ+μ+u2γ,ΓRVTω=∂RVT∂ωωRVT=−ωα+ω+μ+u2γ,ΓRVTσ=∂RVT∂σσRVT=−u1σu1σ+θ+μ.

Similarly, we can compute the sensitivity indexes of *ℛ*_VT_ with respect to the remaining parameters in *ℛ*_VT_, in the same manner. Using the parameter values *γ* = 0.4, *σ* = 1, *η* = 0.35, *ω* = 0.2, *δ* = 0.1, *β* = 0.88, *α* = 0.2, *θ* = 0.0839, and *μ* = 0.02, with *u*_1_ = 0.5 and *u*_2_ = 0.5, the sensitivity indexes of *ℛ*_VT_ are shown in Table 2.

**Figure 3:**
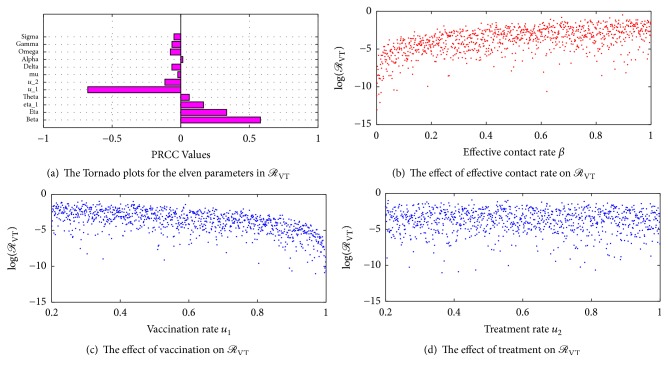
Sensitivity plots.

The corresponding Tornado plots based on a random sample of 1000 points for the twelve parameters in *ℛ*_VT_ are shown in Figure 3(a). The positive values in Table 2 show a promotion of the propagation of the disease. Therefore an increase in the values of *β*, *η*, *η*_1_, *θ*, and *α* will have an increase in the spread of the disease. For example, Γ_*ℛ*_VT__^*β*^ = 1 indicates that increasing the effective contact rate by 10% increases the number of secondary infections by 10%. The negative values in Table 2 indicate a reduction in the effective reproduction number *ℛ*_VT_ if the values of the corresponding parameters are increased. Thus, a reduction in the values of vaccination *u*_1_, treatment *u*_2_, and natural recovery *ω* will lead to an increase in the number of secondary infections in the population.

Figure 3(a) shows the Tornado plots for the twelve parameters in *ℛ*_VT_. It can be seen that, in controlling the spread of bacterial meningitis in a population, more susceptible individuals should be given vaccination. Figure 3(a) also suggests that carriers are likely to have more contacts with the susceptible population than the ill individuals who will typically be bound to their beds during the acute phase of the disease. Therefore, the probability of ill individuals transmitting the infections to susceptibles may be lower than that of carriers who are able to mix well with others within the population. Figures 3(b), 3(c), and 3(d) show the regression plots of effective contact rate (*β*), vaccination rate (*u*_1_), and treatment rate (*u*_2_), respectively.  Figure 3(b) shows that transmission rate has a positive correlation in the spread of bacterial meningitis.  Figure 3(c) shows that vaccination has a negative correlation in the spread of bacterial meningitis and hence vaccination increases the immunity of individuals against the meningitis infection, thereby reducing the spread of the infection.  Figure 3(d) shows that individuals who receive treatment after being infected with bacterial meningitis have a higher chance of recovery and that reduces the spread of the infection and deaths due to bacterial meningitis.

## 3. Optimal Control Problem

Since the goal of this paper is to find the best ways to control the spread of meningitis, we define the following optimal control problem:

(29) J=minu1σ,u2⁡∫0TA1Ct+A2It+B12u12t+B22u22tdt,

subject to model (2).

The admissible control set *U* is Lebesgue measurable, which is defined by

(30) U=u1t,u2t ∣ 0≤u1≤u1max≤1,  0≤u2≤u2max≤1,  t∈0,T.

Our objective is to find (*u*_1_^*∗*^, *u*_2_^*∗*^) ∈ *U* which minimizes the associated cost of the vaccination and the associated cost of the treatment over the specified time interval, as well as minimizing the number of infections at a terminal time (see also [37]). The coefficients *A*_1_ > 0 and *A*_2_ > 0 are constants that are introduced to maintain a balance in the size of *C*(*t*) and *I*(*t*), respectively. *B*_1_ > 0 and *B*_2_ > 0 are the corresponding weights associated with the cost of vaccination (*u*_1_) and treatment (*u*_2_), respectively. The higher bounds (maximum) attainable for the control measures *u*_1_*σ* and *u*_2_ are *u*_1max_ and *u*_2max_, respectively. We fix the control measures *u*_1_ and *u*_2_ to lie between 0 and 1 so that *u*_1max_ = 1 and *u*_2max_ = 1. Therefore the attainment of *u*_1max_ and *u*_2max_ depends on the number of resources available [37]. These resources may include the human effort, material resources, cost of producing vaccine and disbursement, infrastructural resources, the number of health facilities in the community, and the number of hospital beds at the health facilities. The cost of hospitalization, medical test, diagnosis, drug cost, and so forth (see [38–40]) can be associated with treatment. The cost of vaccination may include the cost of the vaccine, the cost of production, the cost of disbursement, the vaccine storage cost, and other related overheads [37]. The severity of the side effects and overdoses of the vaccination and treatment is taken care of by squaring the control measures, and *T* is the final time during the optimal simulation.

### 3.1. Existence of the Optimal Control

Model (2) can be written as

(31) $$\begin{array}{c} G_{t}=K\left(\;G\;\right)+F\left(\;G\;\right), \end{array}$$

where

(32) G=StCtItRt,K=−μ+u1σ00θ0−α+ω+μ+u2γ000α−δ+μ+u1σ0u1σω+u2γu2γ−θ+μ,FG=π−1−u1σβStηCt+η1ItNt1−u1σβStηCt+η1ItNt00,

and *G*_*t*_ is the times derivative of *G*(*t*). System (31) is nonlinear with a bounded coefficient.

Setting *G*_1_ = (*S*_1_(*t*), *C*_1_(*t*), *I*_1_(*t*), *R*_1_(*t*)) and *G*_2_ = (*S*_2_(*t*), *C*_2_(*t*), *I*_2_(*t*), *R*_2_(*t*)) gives

(33) $$\begin{array}{c} F\left(\;G_{1}\;\right)-F\left(\;G_{2}\;\right)=\left[\begin{array}{c} -\left(1-u_{1}\sigma \right)\beta \eta \left(\frac{S_{1}\left(t\right)C_{1}\left(t\right)}{N_{1}\left(t\right)}-\frac{S_{2}\left(t\right)C_{2}\left(t\right)}{N_{2}\left(t\right)}\right)-\left(1-u_{1}\sigma \right)\beta \left(\frac{S_{1}\left(t\right)I_{1}\left(t\right)}{N_{1}\left(t\right)}-\frac{S_{2}\left(t\right)I_{2}\left(t\right)}{N_{2}\left(t\right)}\right)\\ \left(1-u_{1}\sigma \right)\beta \eta \left(\frac{S_{1}\left(t\right)C_{1}\left(t\right)}{N_{1}\left(t\right)}-\frac{S_{2}\left(t\right)C_{2}\left(t\right)}{N_{2}\left(t\right)}\right)-\left(1-u_{1}\sigma \right)\beta \left(\frac{S_{1}\left(t\right)I_{1}\left(t\right)}{N_{1}\left(t\right)}-\frac{S_{2}\left(t\right)I_{2}\left(t\right)}{N_{2}\left(t\right)}\right)\\ 0\\ 0 \end{array}\right]. \end{array}$$

Therefore

(34) FG1−FG2=−1−u1σβηS1tC1tN1t−S2tC2tN2t+−1−u1σβη1S1tI1tN1t−S2tI2tN2t+1−u1σβηS1tC1tN1t−S2tC2tN2t+−1−u1σβη1S1tI1tN1t−S2tI2tN2t≤21−u1σβηS1tC1tN1t−S2tC2tN2t+21−u1σβη1S1tI1tN1t−S2tI2tN2t≤21−u1σβηS1C1−S2C2+21−u1σ·βη1S1I1−S2I2≤21−u1σ·βηC1S1−S2+S2C1−C2+21−u1σ·βη1I1S1−S2+S2I1−I2≤S1−S2·21−u1σβηC1+21−u1σβη1I1+21−u1σβηS2C1−C2+21−u1σ·βη1S2I1−I2≤21−u1σβπμη+1·S1−S2+21−u1σβηπμC1−C2+21−u1σβη1πμI1−I2≤MS1−S2+C1−C2+I1−I2,

where

(35) M=max21−u1σβπμη+1,21−u1σβηπμ,21−u1σβη1πμ,

so that

(36) DG1−DG2≤VG1−G2,where  V=max⁡M,K<∞.

The function *D* is therefore uniformly Lipschitz continuous. From the definition of the control measures *u*_1_(*t*) and *u*_2_(*t*) and the constraint on the state variables, such that *S*(*t*) > 0, *C*(*t*) ≥ 0, *I*(*t*) ≥ 0, and *R*(*t*) ≥ 0, we observe that a solution of system (31) exists [40, 53, 54]. From the objective functional and its associated constraints in model (2), we can find the optimal solution for our model. Firstly, we find the Lagrangian (*L*) and Hamiltonian (*H*) for the control problem [55]. The Lagrangian of the optimal problem is given by

(37) LS,C,I,R,u1,u2=A1Ct+A2It+B12u12t+B22u22t.

Our focus is to find the minimal value of the Lagrangian function, which is done by a pointwise minimization of the Hamiltonian (*H*) defined as follows (using Pontryagin's maximum principle):

(38) HS,C,I,R,u1,u2,t=A1C+A2I+B12u12+B22u22+λ1π−1−u1σβSηC+η1IN−μ+u1σS+θR+λ21−u1σβSηC+η1IN−α+ω+μ+u2γC+λ3αC−δ+μ+u2γI+λ4u2γI+ω+u2γC+u1σS−θ+μR,

where *λ*_*i*_, *i* = 1,2, 3,4, are the adjoint variables associated with *S*(*t*), *C*(*t*), *I*(*t*), and *R*(*t*), defined by

(39) dλ1dt=−∂H∂S,dλ2dt=−∂H∂C,dλ3dt=−∂H∂I,dλ4dt=−∂H∂R.

**Theorem 6 .**

There exists an optimal control pair *u*_1_^*∗*^(*t*), *u*_2_^*∗*^(*t*) such that

(40) $$\begin{array}{c} J\left(\;u_{1}^{*}\left(\;t\;\right),u_{2}^{*}\left(\;t\;\right)\;\right)=\underset{u_{1},u_{2}\in U}{\min}\;J\left(\;u_{1}\left(\;t\;\right),u_{2}\left(\;t\;\right)\;\right). \end{array}$$

**Proof**

We start our proof by considering the properties of the existence of the optimal control (see [56]). Following [57], the set of control measures with corresponding state variables are positive. The set *U* is convex and closed by definition. Therefore, our optimal system is closed and bounded which ascertains the compactness required for the existence of the optimal control. Additionally, the integrand in the objective functional (29), *A*_1_*C*(*t*) + *A*_2_*I*(*t*) + (*B*_1_/2)*u*_1_^2^(*t*) + (*B*_2_/2)*u*_2_^2^(*t*), is convex on the control set *U*. Furthermore, we can state that there exists a positive constant *ρ* > 1 [58], and nonnegative numbers *ν*_1_ and *ν*_2_ such that the objective functional has a lower bound of *ν*_1_(|*u*_1_|^2^ + |*u*_2_|^2^)^*ρ*/2^ − *ν*_2_ so that

(41) $$\begin{array}{c} J\left(\;u_{1}\left(\;t\;\right),u_{2}\left(\;t\;\right)\;\right)\geq \nu_{1}{\left(\;{\left|\;u_{1}\;\right|}^{2}+{\left|\;u_{2}\;\right|}^{2}\;\right)}^{\rho /2}-\nu_{2}, \end{array}$$

since the control measures and the state variables are bounded, this leads us to a compact proof of existence of the optimal control.

### 3.2. Characterization of the Optimal Control

We will apply Pontryagin's maximum principle to the Hamiltonian function above to derive the necessary condition of optimality for our control problem.

**Theorem 7 .**

Let *S*, *C*, *I*, and *R* be optimal state solutions with corresponding optimal control variables *u*_1_^*∗*^ and *u*_2_^*∗*^ for the objective functional and its constraints in model (2) with *N* = *S* + *C* + *I* + *R*. Then, there exist four adjoint variables *λ*_1_, *λ*_2_, *λ*_3_, and *λ*_4_ that satisfy

(42) dλ1dt=λ11−u1σβηC+η1IN−1−u1σβSηC+η1IN2+μ+u1σ+λ21−u1σβSηC+η1IN2−1−u1σβηC+η1IN−λ4u1σ,dλ2dt=−A1+λ11−u1σβηSN−1−u1σβSηC+η1IN2+λ2α+ω+μ+u2γ+λ21−u1σβSηC+η1IN2−1−u1σβηSN−λ3α−λ4ω+u2γ,dλ3dt=−A2+λ11−u1σβη1SN−1−u1σβSηC+η1IN2+λ21−u1σβSηC+η1IN2−1−u1σβη1SN+λ3δ+μ+u2γ−λ4u2γ,dλ4dt=−λ1θ+λ4θ+μ,

with transversality conditions

(43) λiT=0,i=1,2,3,4for  the  control  set  U=u1,u2,  such  that  ∂H∂ui=0,where  i=1,2.

Therefore, the optimal control pair (*u*_1_^*∗*^, *u*_2_^*∗*^) is given by

(44) u1∗=min⁡max⁡0,1B1βSηC+η1IS+C+I+Rλ1−λ2+λ1−λ4σS,u1max,u2∗=min⁡max⁡0,γλ2−λ4C+γλ3−λ4IB2,u2max.

**Proof**

We use the Hamiltonian function in (38) in order to obtain the adjoint relations and the transversality conditions. We set the state variables in the Hamiltonian function to *S*, *C*, *I*, and *R*, and differentiating the Hamiltonian (*H*) with respect to *S*, *C*, *I*, and *R*, respectively, yields (42). Also, differentiating the Hamiltonian (*H*) with respect to the control measures *u*_1_*σ* and *u*_2_ in the interior of *U*, we obtain the optimality conditions below:

(45) ∂H∂u1=B1u1−λ1βSηC+η1IS+C+I+R−λ1σS+λ2βSηC+η1IS+C+I+R+λ4σS=0,∂H∂u2=B2u2−λ2γC−λ3γI+λ4γC+λ4γI=0.

Plugging *u*_1_ = *u*_1_^*∗*^ and *u*_2_ = *u*_2_^*∗*^ into (45) and solving the optimal control pair (*u*_1_^*∗*^, *u*_2_^*∗*^), we have

(46) u1∗=1B1βSηC+η1IS+C+I+Rλ1−λ2+λ1−λ4σS,u2∗=γλ2−λ4C+γλ3−λ4IB2.

The two control measures which are bounded with lower bounds zero and upper bounds *u*_*i*max_ = 1, where *i* = 1,2, give

(47) u1∗∈U⟹u1∗t=0if  1B1βSηC+η1IS+C+I+Rλ1−λ2+λ1−λ4σS≤0,1B1βSηC+η1IS+C+I+Rλ1−λ2+λ1−λ4σSif  0<1B1βSηC+η1IS+C+I+Rλ1−λ2+λ1−λ4σS<u1max,u1maxif  1B1βSηC+η1IS+C+I+Rλ1−λ2+λ1−λ4σS≥u1max,u2∗∈U⟹u2∗t=0if  γλ2−λ4C+γλ3−λ4IB2≤0,γλ2−λ4C+γλ3−λ4IB2if  0<γλ2−λ4C+γλ3−λ4IB2<u2max,u2maxif  γλ2−λ4C+γλ3−λ4IB2≥u2max.

Using (47), the optimal control measures are characterized as (44), completing the proof.

Therefore, our optimality system is given by

(48) dSdt=π−1−minmax0,1B1βSη1I+ηCS+C+I+Rλ1−λ2+λ1−λ4σS,u1maxβSηC+η1IN−μ+minmax0,1B1βSηC+η1IS+C+I+Rλ1−λ2+λ1−λ4σS,u1maxσS+θR,dIdt=αC−δ+μ+minmax0,γλ2−λ4C+γλ3−λ4IB2,u2maxγI,dRdt=minmax0,γλ2−λ4C+γλ3−λ4IB2,u2maxγI+ω+minmax0,γλ2−λ4C+γλ3−λ4IB2,u2maxγC−θ+μR+minmax0,1B1βSηC+η1IS+C+I+Rλ1−λ2+λ1−λ4σS,u1max

(49) dλ1dt=λ11−minmax0,1B1βSηC+η1IS+C+I+Rλ1−λ2+λ1−λ4σS,u1maxβηC+η1IN−λ11−minmax0,1B1βSηC+η1IS+C+I+Rλ1−λ2+λ1−λ4σS,u1maxβSηC+η1IN2+λ1μ+minmax0,1B1βSηC+η1IS+C+I+Rλ1−λ2+λ1−λ4σS,u1maxσ+λ21−minmax0,1B1βSηC+η1IS+C+I+Rλ1−λ2+λ1−λ4σS,u1maxβSηC+η1IN2−λ21−minmax0,1B1βSηC+η1IS+C+I+Rλ1−λ2+λ1−λ4σS,u1maxβηC+η1IN−λ4minmax0,1B1βSηC+η1IS+C+I+Rλ1−λ2+λ1−λ4σS,u1maxσ,

(50) dλ2dt=−A1+λ11−minmax0,1B1βSηC+η1IS+C+I+Rλ1−λ2+λ1−λ4σS,u1maxβηSN−λ11−minmax0,1B1βSηC+η1IS+C+I+Rλ1−λ2+λ1−λ4σS,u1maxβSηC+η1IN2+λ2α+ω+μ+minmax0,γλ2−λ4C+γλ3−λ4IB2,u2maxγ+λ21−minmax0,1B1βSηC+η1IS+C+I+Rλ1−λ2+λ1−λ4σS,u1maxβSηC+η1IN2−λ21−minmax0,1B1βSηC+η1IS+C+I+Rλ1−λ2+λ1−λ4σS,u1maxβηSN−λ3α−λ4ω+minmax0,γλ2−λ4C+γλ3−λ4IB2,u2maxγ,dλ3dt=−A2+λ11−minmax0,1B1βSηC+η1IS+C+I+Rλ1−λ2+λ1−λ4σS,u1maxβη1SN−λ11−minmax0,1B1βSηC+η1IS+C+I+Rλ1−λ2+λ1−λ4σS,u1maxβηC+η1IN2+λ21−minmax0,1B1βSηC+η1IS+C+I+Rλ1−λ2+λ1−λ4σS,u1maxβηC+η1IN2−λ21−minmax0,1B1βSηC+η1IS+C+I+Rλ1−λ2+λ1−λ4σS,u1maxβη1SN+λ3δ+μ+minmax0,γλ2−λ4C+γλ3−λ4IB2,u2maxγ−λ4minmax0,γλ2−λ4C+γλ3−λ4IB2,u2maxγ,dλ4dt=−λ1θ+λ4θ+μ,with  λiT=0,  i=1,2,3,4

## 4. Numerical Simulations of the Optimal Control

**Figure 4:**
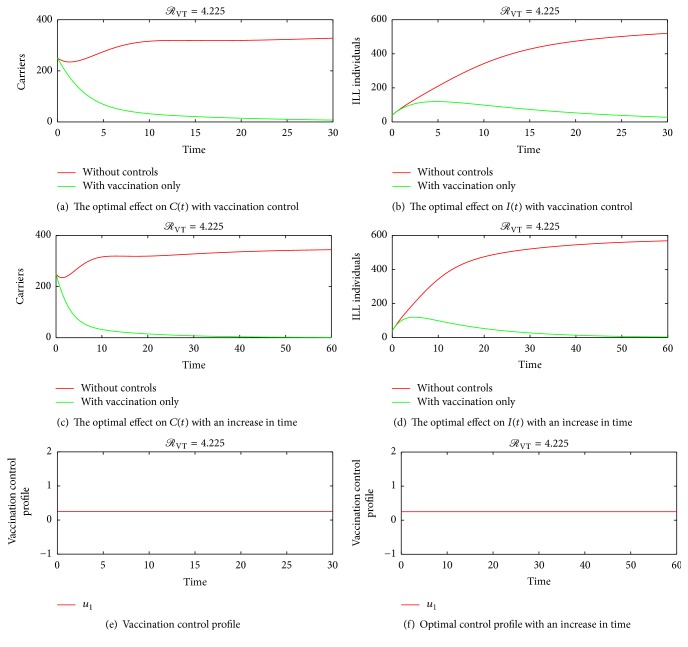
(a), (b), (c), and (d) show that administering vaccination on susceptible individuals reduces the number of secondary infections in the carrier and the ill population. (e) and (f) show the optimal control profile of vaccination. Initial conditions *S*(0) = 700, *C*(0) = 250, *I*(0) = 40, and *R*(0) = 10 and the parameter values *π* = 100, *η* = 0.35, *ω* = 0.2, *δ* = 0.1, *σ* = 1, *α* = 0.2  and  *θ* = 0.0839 were used in this simulation.

**Figure 5:**
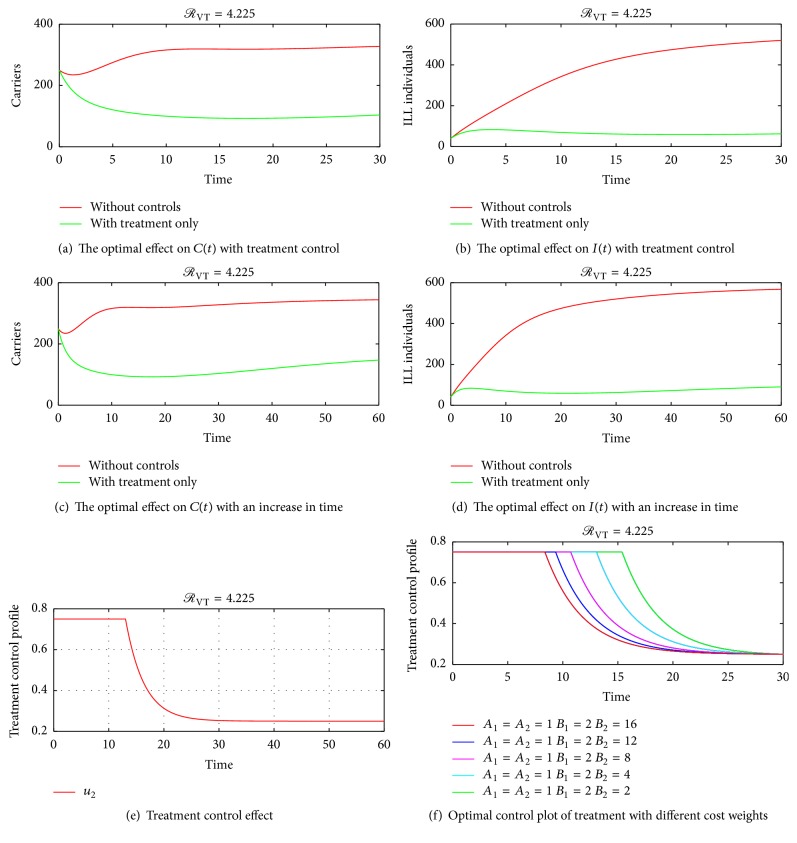
(a) and (b) show that giving treatment to carriers and the ill individuals reduces the number of secondary infections in the carrier and the ill population but the disease may not be eradicated if the use of only treatment is stuck to. Furthermore, we increased time so as to see whether the disease could be eradicated when using treatment only. We see in (c) and (d) that there is a possibility of recurrence of the disease using only treatment control strategy as indicated by the curved green line in (c) and the linear flow of the green line in (d). (e) shows that, to minimize bacterial meningitis outbreak in 30 days, the treatment control should be held intensively for 30 days at a constant rate. (f) shows the optimal control plot of treatment with different cost weights.

**Figure 6:**
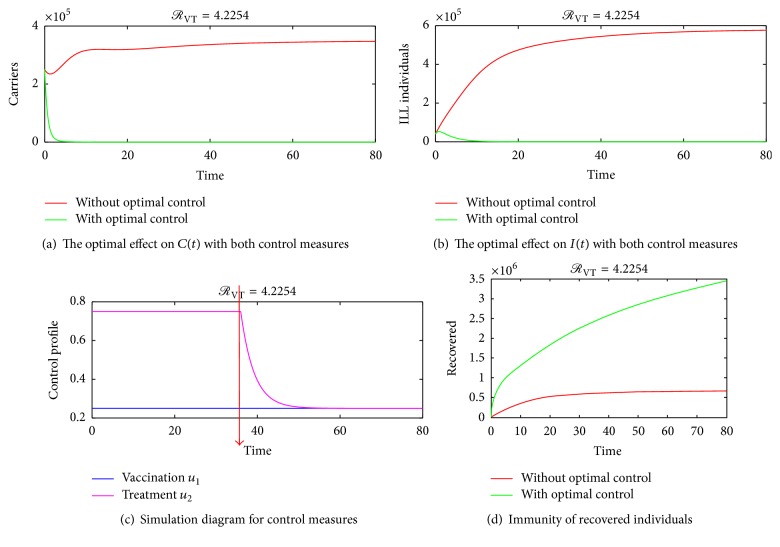
Further simulation trajectories. (d) shows the immunity levels when the two control measures are applied with the following parameter values: *π* = 100000, *η* = 0.35, *ω* = 0.2,  *δ* = 0.1, *σ* = 1, *α* = 0.2, and *θ* = 0.0839.

The optimality system consisting of the state equations (2) and the adjoint equations (45) is solved using the forward-backward sweep scheme. The solution is started with an initial guess for the control measures and the final time set to *T* = 30 days and later varied to *T* = 60 days and *T* = 80 days. The state system is solved forward in time while the costate system is solved backward in time. The current solutions of the state system together with the initial guess for the control measures *u*_1_ and *u*_2_ are used to solve the costate system. The controls *u*_1_ and *u*_2_ are updated using the characterizations in (47). With the current cosystem solution and updated controls, the state system is solved and the whole solution process continues until convergence is achieved. Since bacterial meningitis is an endemic disease especially in Sub-Sahara Africa, parameter values that make effective reproduction number *ℛ*_VT_ more than unity are considered. Taking the associated costs on carriers and ill individuals as *A*_1_ = *A*_2_ = 1, *B*_1_ = 2, and *B*_2_ = 4, the graphical results of the model are as follows. Figures 4 and 5, show the dynamics of the infection in the presences of control measures and without control measures in a more localised form.  Figure 5(f) shows the effect of varying the cost associated with the treatment control. It is seen that if the weight of treatment cost *B*_2_ is continuously increased, the upper bound time reduces to about 9 days as indicated by the red line in Figure 5(f). This is true because a higher cost of treatment causes the use of the treatment control to be less.  Figure 6 shows the spreading rate of meningitis with and without control measures in a highly dense population, thus in colleges, prisons, cities, and so forth, which suggests that when carriers become more in a highly populated settings there is a likelihood of the infection spreading faster, thereby leading to a corresponding increase in the number of ill (sick) individuals; hence the trajectories in Figures 6(a) and 6(b) show that there is a significant difference in the number of carriers and infected individuals with and without control measures. Therefore, applying both vaccination and treatment has a higher rate of controlling bacterial meningitis than depending only on one control method. Figures 6(a) and 6(b) also show that as infections increase in the population, there is a need for introducing vaccination and treatment at the earlier stage so as to minimize the spread of the infection in the population. This further suggests that college students and prisoners should be given regular vaccination against meningitis, since bacterial meningitis is more likely to affect college students and prisoners than other people, due to the close proximate of beds in the dormitories and prison cells, especially in Sub-Saharan Africa.  Figure 6(c) shows that the trajectory for treatment moves to an upper bound at about *t* = 35 days and slowly decreases to a lower bound, which implies that, during the period before 35 days, substantial amount of the control *u*_2_ should be applied while administering the vaccination control in the susceptible population so as to reduce the number of carriers and ill individuals until the 50th day. Afterwards, less amount of treatment can be used since the number of carriers and ill individuals will be considerably reduced by the earlier investment in treatment within the period before 35 days.

**Figure 7:**
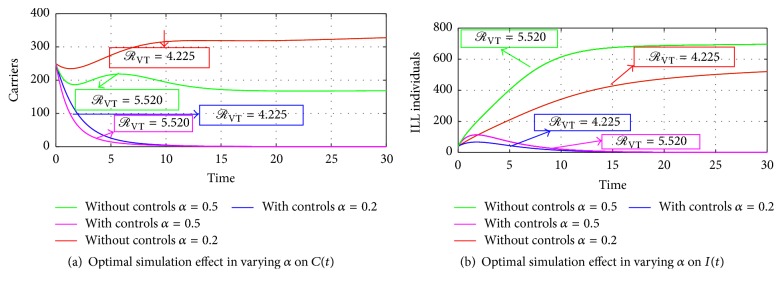
Simulation results of the optimal control model with *α* = 0.2-0.5, on carriers and the ill compartment.

**Figure 8:**
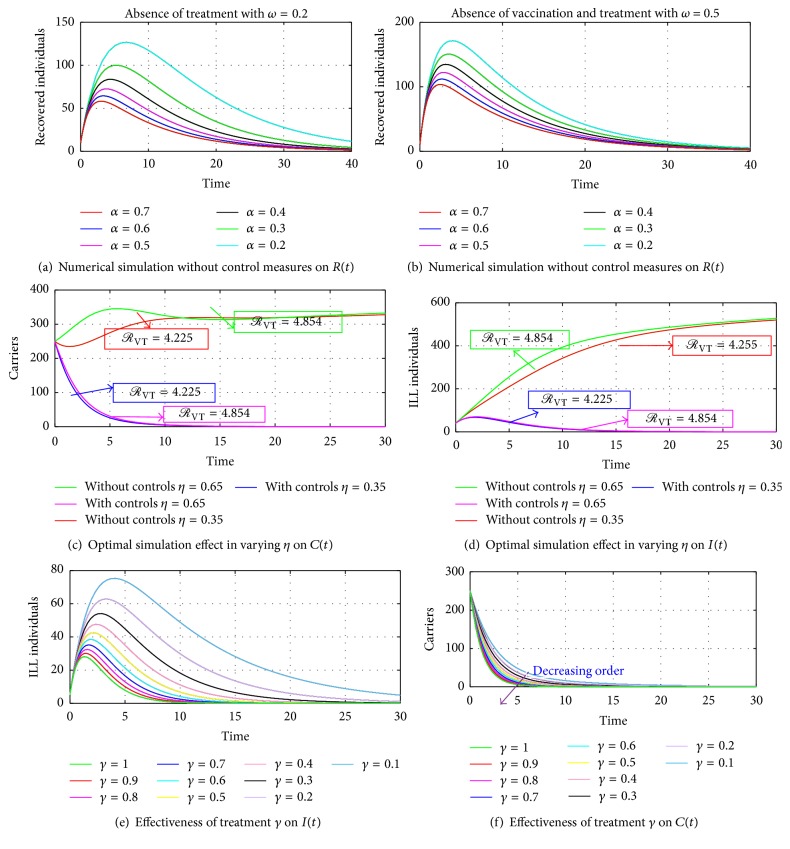
Simulation results of the optimal control model with *η* = 0.35-0.65. The solutions for the carriers and ill individuals with *α* = 0.2 and *α* = 0.5 give *ℛ*_VT_ = 4.225 and *ℛ*_VT_ = 5.250, respectively. The case where *η* = 0.35 and *η* = 0.65 gives *ℛ*_VT_ = 4.225 and *ℛ*_VT_ = 4.854, respectively, which indicates the existence of an unstable disease-free equilibrium and a stable endemic equilibrium. In (e) and (f), vaccination and treatment rates are set to 0.5 and the effectiveness of the treatment control *γ* varied. (e) and (f) show that as the effectiveness of the treatment control increases, the number of ill individuals reduces faster as compared to the number of carriers.

Figures 7(a), 7(b), 8(c), and 8(d) show the impact of varying *α* and *η*. It can be seen that an increase in *α* and *η* without control measures has a corresponding decrease in the number of carriers and a considerable increase in the number of infected individuals. Therefore, applying the control measures gives a drastic decrease in the number of infections in both carriers and the ill compartment at a higher rate of *α* = 0.5 and *η* = 0.65. Furthermore, in Figures 7(a) and 7(b), authors kept the natural recovery rate (*ω*) fixed at 0.2 while assessing the effect of varying the rate of falling ill on carriers and on ill individuals, respectively. Hence, considering an increased rate from 0.2 to 0.7 of the rate of falling ill as shown in Figures 8(a) and 8(b) demonstrates how serious the infection is in the absence of vaccination or treatment, which indicates that ill individuals have a lower means of recovering from the disease, not even through a higher rate of natural defences of *ω* = 0.5 as indicated in Figure 8(b) without vaccination and treatment, which clearly shows that vaccination and treatment are essential in controlling meningitis irrespective of the age group; therefore we suggest that health authorities should increase the rate at which susceptible individuals get vaccination through public education and also encourage individuals with and without symptoms of the infection to visit health centers for a quick check-up and if the infection is detected an immediate treatment/vaccination should be given on time to avert the spreading of the infection to community members, relatives, or close friends.

### 4.1. Vaccination Control Only

Figures 4(a)–4(f) show simulation results of implementing only control strategy *u*_1_ with *u*_2_ set to zero.

### 4.2. Treatment Control Only

Considering treatment control, *u*_2_, by setting the vaccination control, *u*_1_, to zero, the simulation results are as shown in Figures 5(a)–5(f).

### 4.3. Applying Both Control Measures

Finally, both control measures are implemented using the following initial population size of *S*(0) = 700000, *C*(0) = 250000, *I*(0) = 40000, and *R*(0) = 10000 and the results are plotted in Figures 6(a)–6(d).

**Figure 9:**
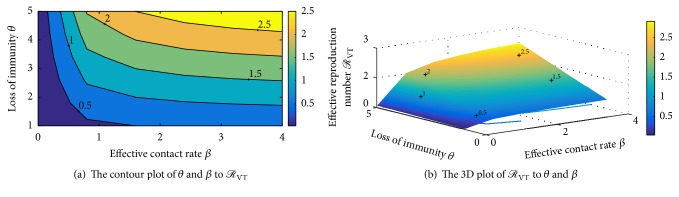
The interepidemic period of the SCIRS model depending on parameters *θ* and *β*.

**Figure 10:**
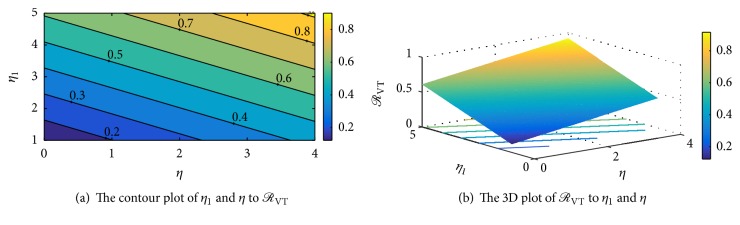
The interepidemic period of the* SCIRS* model depending on parameters *η* and *η*_1_.

Figures 9(a), 9(b), 10(a), and 10(b) show the interepidemic nature of some selected parameters in the *SCIRS* model presented above. Figures 9(a) and 9(b) indicate that as individuals lose immunity and become susceptible again, there is a corresponding increase in the rate of infection, and this suggests that an individual who receives vaccination and/or treatment and remains immune for 3–5 years should immediately go for another vaccination against the infection so as to avert transmission of the infection to that individual. Figures 10(a) and 10(b) depict that the per capita infection rate by ill individuals, *η*_1_, and the per capita infection rate by carriers, *η*, are interlinked and so have a corresponding influence in the spread of the infection.  Figure 10(b) also indicates that as the infectivity rate of carriers and ill individuals goes beyond 0.6, there could be a high spread of the infection in the community/society should there be an outbreak, since the effective reproduction number will be getting closer to one (1) with a possibility of going beyond a unit.

## 5. Conclusion

We presented a mathematical framework of vaccination and treatment on *SCIRS* bacterial meningitis model. The investigation of the stability of the model shows that the disease-free equilibrium is locally and globally asymptotically stable and the endemic equilibrium depicts a global stability. Scatter plots and the Tornado plots of the twelve parameters in *ℛ*_VT_ show that the effective contact rate *β* has a major impact in transmitting the disease, followed by the infectivity potential of carriers *η*. This supports the fact that asymptomatic carriers are likely to have more contacts in a community/nation when there is an outbreak of bacterial meningitis compared to ill individuals who will typically be bound to their beds during the acute phase of the disease, thereby lowering the probability of infecting susceptible individuals. Finally, the numerical simulation shows that the optimal (best) way of controlling the transmission of meningitis in Sub-Saharan Africa and the world at large is to encourage susceptible individuals to go for vaccination against meningitis at the health centers and also report any suspected symptoms of meningitis to health practitioners for early detection and immediate care (treatment).

## Figures and Tables

**Table 1:** Description of parameter values used in the model. “Due to lack of relevant data, most of the parameter values are assumed within realistic ranges for a typical scenario in a rural community/society for the purpose of illustration” [44], with most of the parameter values where appreciable per year.

Parameter	Description	Value/Range	Reference
*π*	Recruitment rate (migration and/or birth rate)	100–100000	Assumed
*β*	Effective contact rate	0.88	[28]
*θ*	Loss of immunity	0.04–2	[14, 47]
*μ*	Natural death rate	0.02	[14]
*δ*	Disease-induced mortality	0.05–0.5	[48]
*α*	Rate of progression from *C* to *I*	0.1–0.52	[14]
*ω*	Natural recovery rate	0.06–0.2	Assumed
*η*_1_	Per capita infection rate by ill individuals	0.2–0.95	Assumed
*η*	Per capita infection rate by Carriers	0.2–0.85	Assumed
*u*_1_	Vaccination rate of susceptible individuals against Meningitis	[0,1]	Assumed
*u*_2_	Treatment rate for carriers without natural recovery and ill individuals	[0,1]	Assumed
*σ*	Effectiveness of vaccination	0.85–1	[2]
*γ*	Effectiveness of Treatment	0.1–0.9	Assumed

**Table 2:** Sensitivity indexes of *ℛ*_VT_ to the parameters in (6).

Parameter	Description	Sensitivity index
*β*	Effective contact rate	1
*η*	Per capita infection rate by Carriers	0.6154
*η*_1_	Per capita infection rate by ill individuals	0.3846
*θ*	Loss of immunity	0.6686
*α*	Rate of falling ill	0.0620
*μ*	Natural death rate	0.1031
*δ*	Disease-induced death rate	−0.1202
*ω*	Natural recovery rate	−0.3226
*u*_1_	Vaccination rate	−1.8280
*σ*	Effectiveness of vaccination	−0.8280
*u*_2_	Treatment rate	−0.5630
*γ*	Effectiveness of treatment	−0.5230
